# Developmental Accumulation of Gene Body and Transposon Non-CpG Methylation in the Zebrafish Brain

**DOI:** 10.3389/fcell.2021.643603

**Published:** 2021-03-04

**Authors:** Samuel E. Ross, Daniel Hesselson, Ozren Bogdanovic

**Affiliations:** ^1^Genomics and Epigenetics Division, Garvan Institute of Medical Research, Sydney, NSW, Australia; ^2^Faculty of Medicine, St Vincent’s Clinical School, University of New South Wales, Sydney, NSW, Australia; ^3^Centenary Institute, The University of Sydney, Sydney, NSW, Australia; ^4^Faculty of Medicine and Health, The University of Sydney, Sydney, NSW, Australia; ^5^School of Biotechnology and Biomolecular Sciences, University of New South Wales, Sydney, NSW, Australia

**Keywords:** DNA methylation, brain, nervous system, zebrafish, repetitive elements

## Abstract

DNA methylation predominantly occurs at CG dinucleotides in vertebrate genomes; however, non-CG methylation (mCH) is also detectable in vertebrate tissues, most notably in the nervous system. In mammals it is well established that mCH is targeted to CAC trinucleotides by DNMT3A during nervous system development where it is enriched in gene bodies and associated with transcriptional repression. Nevertheless, the conservation of developmental mCH accumulation and its deposition by DNMT3A is largely unexplored and has yet to be functionally demonstrated in other vertebrates. In this study, by analyzing DNA methylomes and transcriptomes of zebrafish brains, we identified enrichment of mCH at CAC trinucleotides (mCAC) at defined transposon motifs as well as in developmentally downregulated genes associated with developmental and neural functions. We further generated and analyzed DNA methylomes and transcriptomes of developing zebrafish larvae and demonstrated that, like in mammals, mCH accumulates during post-embryonic brain development. Finally, by employing CRISPR/Cas9 technology, we unraveled a conserved role for Dnmt3a enzymes in developmental mCAC deposition. Overall, this work demonstrates the evolutionary conservation of developmental mCH dynamics and highlights the potential of zebrafish as a model to study mCH regulation and function during normal and perturbed development.

## Background

In genomes of vertebrate adult somatic cells, the majority of CpG sites are methylated (>80%) with the exception of CpG-rich promoters and distal regulatory elements (Bird, 2002; Jones, 2012; Schübeler, 2015). While otherwise ubiquitous, CpG methylation (mCG) at regulatory elements is known to participate in long-term gene silencing processes (de Mendoza et al., 2019). In mammals, albeit at much lower levels, methylation of cytosines outside the CpG context (mCH, H = T,C,A) has also been reported in the majority of tissues (Schultz et al., 2015). mCH, or more particularly methylation of CA dinucleotides (mCA), occurs most commonly in mammalian embryonic stem cells (ESCs) and in the brain (Lister et al., 2009, 2013; Schultz et al., 2015). In ESCs, mCH is enriched at CAG trinucleotides in gene bodies and is positively correlated with gene expression. Additionally, increased levels of mCH were observed at repetitive elements in ESCs (Ziller et al., 2011; Arand et al., 2012; Guo et al., 2014b). In mammalian brains, mCH rivals the levels of mCG and is enriched at CAC trinucleotides (mCAC) in gene bodies where it negatively correlates with expression and is deposited *de novo* by DNMT3A (Lister et al., 2013). In line with its repressive role in the nervous system, mCH is depleted at open chromatin regions (Lister et al., 2013). mCH in the postnatal mammalian brain displays a rapid increase during initial phases of synaptogenesis, which corresponds to 2–4 weeks in mouse, and first 2 years of life in humans. This is followed by a longer period of slower accumulation (Lister et al., 2013). While mCH is found at high levels and studied extensively in plants (Zhang et al., 2018), the function of mCH in vertebrates is less well known. Several studies, however, have demonstrated that Methyl-CpG Binding Protein 2 (MeCP2) is able to bind to and regulate genes marked by mCH, which was particularly evident at long genes (Guo et al., 2014a; Chen et al., 2015; Gabel et al., 2015; Boxer et al., 2020; Clemens et al., 2020). Whether this is due to biological or technical reasons is currently debated (Raman et al., 2018). Mutations in MeCP2 are the most prevalent cause of Rett syndrome, and interestingly, altered readout of mCH deposited by DNMT3A appears to play a central role in Rett syndrome pathogenesis (Chen et al., 2015; Lavery et al., 2020). MeCP2 is conserved across vertebrates, such as zebrafish, where depletion of MeCP2 results in similar pathologies to Rett syndrome including altered motor behavior, improper synapse formation and acute inflammation (Pietri et al., 2013; Gao et al., 2015; Nozawa et al., 2017; van der Vaart et al., 2017).

A recent report described the conserved enrichment of mCH in vertebrate brains, which originated alongside MeCP2 and DNMT3A enzymes at the root of the vertebrate lineage (de Mendoza et al., 2021). This study also highlighted the anti-correlation between gene body mCH and expression in some, but not all, vertebrate brains. In our previous work, we found highly specific mCH enrichment at TGCT tetranucleotides within zebrafish mosaic satellite repeats in embryonic and adult tissues, deposited by the teleost specific Dnmt3ba enzyme (Ross et al., 2020). However, the developmental dynamics and distribution of neural-specific mCH, and a functional role for DNMT3A or MeCP2 in relation to mCH, has yet to be demonstrated outside of mammalian brains. Here we expand upon the utility of the zebrafish model in the study of mCH and reveal that like in mammals, mCH accumulates during brain development via Dnmt3a enzymes and becomes enriched at downregulated genes and Tc1-like transposable elements. This study thus extends our knowledge of vertebrate mCH conservation and lays the foundation for future work that will allow for the precise dissection of mCH regulatory functions during zebrafish embryogenesis and nervous system formation.

## Materials and Methods

### Zebrafish Usage and Ethics

Zebrafish work was conducted at the Garvan Institute of Medical Research in accordance with the Animal Ethics Committee AEC approval and with the Australian Code of Practice for Care and Use of Animals for Scientific Purposes. Adult wild type (AB/Tübingen) *Danio rerio* (zebrafish) were bred in an equal ratio of males and females. Embryos were collected 0 h post-fertilization (hpf) and incubated in 1X E3 medium (5 mM NaCl, 0.33 mM CaCl2, 0.17 mM KCl, 0.33mM H14MgO11S) for 4 days at 28.5°C before being transferred onto a filtered system.

### Genomic DNA and RNA Extraction

Whole brains were dissected from zebrafish larvae and adults before being snap-frozen in liquid nitrogen and stored at −80°C. Genomic DNA (gDNA) was extracted from brains using the QIAGEN DNeasy Blood & Tissue Kit (QIAGEN, Chadstone, VIC, Australia) according to manufacturer’s instructions. For RNA extraction, half of the lysate from the first step of DNA extraction from the QIAGEN DNeasy Blood & Tissue Kit was added to TRIsure (Bioline) and purified following manufacturer’s instructions. All experiments in this study were performed in two biological replicates.

### CRISPR/Cas9 Zebrafish Knockouts

Guide RNAs (gRNA) targeting *dnmt3aa* and *dnmt3ab* loci were designed with CRISPRscan (Moreno-Mateos et al., 2015). gRNAs for both loci were synthesized and co-injected into 1-cell stage embryos as previously described (Ross et al., 2020). CRISPR/Cas9 knockouts (cKO) fish were grown to 4 weeks of age before their brains were harvested for DNA and RNA extraction. Amplicons surrounding the CRISPR/Cas9 cut sites were PCR-amplified from genomic DNA, ligated to NEXTFLEX Bisulfite-Seq barcodes (PerkinElmer, Waltham, MA, United States), and spiked into libraries that were sequenced on the Illumina HiSeq X platform. Knockout efficiencies were calculated from the sequenced amplicons using CRISPResso (Pinello et al., 2016). RNA was reverse transcribed to cDNA using the SensiFAST^TM^ cDNA Synthesis Kit (Bioline), following the manufacturer’s protocol and subjected to qPCR analysis. Relative expression levels were calculated using the 2−ΔΔCT method and *bactin* gene as the control transcript. Two sample *t*-tests were performed on CT values. All oligos used in this study can be found in Supplementary Table 1.

### Whole Genome Bisulfite Sequencing (WGBS)

WGBS libraries were prepared from 500 ng of zebrafish brain gDNA, spiked with 0.025 ng of unmethylated lambda phage DNA (Promega, Madison, WI, United States) and sequenced on the Illumina HiSeq X platform (2 × 150 bp) as previously described (Ross et al., 2020).

### Reduced Representation Bisulfite Sequencing (RRBS)

RRBS libraries were prepared from 500 ng of zebrafish brain gDNA spiked with 0.025 ng of unmethylated lambda phage DNA (Promega, Madison, WI, United States). gDNA was digested with 10 U *Bcc*I (CCATC(N)_4_) and 10 U *Ssp*I (AATATT) for 2 h. A separate aliquot was digested with 20 U *Msp*I (New England BioLabs, Ipswich, MA, United States). RRBS libraries were constructed as previously described (Ross et al., 2020), sequenced, and the BAM files corresponding to different digestion reactions (*Bcc*I/*Ssp*I or *Msp*I) were merged before downstream analysis.

### WGBS and RRBS Data Analyses

WGBS reads were trimmed with Trimmomatic: ILLUMINACLIP:TruSeq3-PE.fa:2:30:10 SLIDINGWINDOW: 5:20 LEADING:3 TRAILING:3 MINLEN:60 (Bolger et al., 2014), and mapped using WALT (-m 5 -t 20 -N 10000000) (Chen et al., 2016) onto the GRCz11 reference genome (UCSC), containing the λ genome. BAM files, containing only uniquely mapped reads, were deduplicated using sambamba markdup (Tarasov et al., 2015). RRBS data were processed as above with the additional option of: HEADCROP:5 CROP:140 added during trimming and without deduplication. BAM files were made FLAG-compatible and processed with CGmapTools (Chen et al., 2016; Guo et al., 2018) (convert bam2cgmap) to obtain ATCGmap files, which were corrected for CH positions that showed evidence of CG SNPs (de Mendoza et al., 2021). A summary of library statistics can be found in Supplementary Table 2. Genomic data were visualized in UCSC (Kent et al., 2002) and IGV (Robinson et al., 2011) browsers.

### DNA Sequence Motif Analyses

BED file coordinates of the 10,000 most highly methylated mCH and mCAC sites, with a minimal depth of 10, were extended by 4 base pairs upstream and downstream. The resulting files were used as input for HOMER “findMotifsGenome.pl” function (Heinz et al., 2010), establishing the search for *de novo* motifs of length 9 (-len 9 -size given) with the GRCz11 genome used as the background sequence. Motifs were visualized using the “ggseqlogo” package in R (Wagih, 2017) and motif positions in the genome were called using the scanMotifGenomeWide.pl function (with and without -mask option checked).

### mCH Level Calculation and Plotting

Bedgraphs were generated from corrected CGmapTools outputs and converted to bigWig using bedGraphToBigwig script from Kent utils. Average mCH levels were determined from bedGraph files and calculated by dividing the sum of reads supporting a methylated cytosine by the sum of all reads mapping to that position. mCH levels in genomic features and gene bodies were calculated using BEDtools map (Quinlan and Hall, 2010). mCH levels, TPMs, and gene length were plotted using the boxplot function in R (outline = FALSE). Heatmaps were generated using deepTools (Ramirez et al., 2014) computeMatrix with the following parameters: “computeMatrix scale-regions -m 650 -b 500 -a 500 -bs 25.” NAN values were replaced with 0. Heatmaps were plotted with the plotHeatmap function, sorted, and clustered based on methylation levels. Scatterplots were generated using the *geom_bin2d* function in *ggplot2* [(bins = 50) + geom_smooth(method = l m)]. Pearson correlations were calculated using the *rcorr* function in R.

### DMR Calling

Differentially methylated regions (DMRs) were called using DSS (delta = 0.1, p.threshold = 0.05, minlen = 100, minCG = 5, dis.merge = 500, pct.sig = 0.5) (Feng et al., 2014).

### Repeatmasker Track Analyses

Repeatmsker tracks were obtained from UCSC. The percentage of repeat subfamilies overlapping the top-methylated CAC motifs was determined with BEDtools (intersectBed).

### Correlation of mCH With Genomic Features

Sequenced ChIP-seq reads (Kaaij et al., 2016; Yang et al., 2020) were trimmed with Trimmomatic (ILLUMINACLIP:TruSeq3-SE.fa:2:30:10 SLIDINGWINDOW:5:20 LEADING:3 TRAILING:3 MINLEN:20) (Bolger et al., 2014) before being mapped to the GRCz11 genome using Bowtie2 with default settings (Langmead and Salzberg, 2012). BAM files were deduplicated using sambamba markdup (Tarasov et al., 2015). Peaks were called using MACS2 (Zhang et al., 2008). bigWigs were generated using deepTools (Ramirez et al., 2014) bamCompare (-e 300 -p 20 –normalizeUsing RPKM –centerReads), and bedGraphs using UCSC bigWigToBedGraph. Spearman correlations were calculated using BEDtools map (Quinlan and Hall, 2010) and rcorr(type = c(“spearman”) from the *Hmisc* package in R.

### RNA-Seq

RNA-seq libraries were prepared with 1,000 ng of input RNA using the KAPA mRNA HyperPrep Kit, according to manufacturer’s instructions and sequenced on the Illumina HiSeq X platform (2 × 150 bp).

### RNA-Seq Analyses

RNA-seq reads were trimmed using Trimmomatic: ILLUMINACLIP: TruSeq3-PE.fa:2:30:10 SLIDING-WINDOW:5:20 LEADING:3 TRAILING:3 MINLEN:60 (Bolger et al., 2014) and aligned to the GRCz11 genome using STAR (Dobin et al., 2013). Differential gene expression analysis was performed using edgeR (Robinson et al., 2010). Robinson et al., 2010 with genes selected based on a minimum ± 1.5 logFC (FDR < 0.05) between any of the analyzed time points. Z-scores were calculated based on the log2 transformations of TPM values and plotted with the *pheatmap* package in R. Analysis of published RNA-seq data was performed based on the provided read count tables with TPM values calculated from the average of 5–6 month old brain datasets (Aramillo Irizar et al., 2018) or from collated counts of 30 neurons (Lange et al., 2020).

## Results and Discussion

### mCH Is Enriched at Defined CAC-Containing Motifs in Zebrafish Brains

To investigate mCH in the zebrafish nervous system, we analyzed WGBS data (bisulfite conversion > 99.5%) of adult brain (mean coverage = 9.9X), as well as of adult liver (mean coverage = 7.6X), to use as a non-neural control tissue (Bogdanovic et al., 2016; Skvortsova et al., 2019). We employed stringent genotype correction (de Mendoza et al., 2021) to allow for more sensitive interrogation of mCH patterns. To better understand the sequence context of mCH deposition in the zebrafish brain and how it compares to non-neural tissues (liver), we performed a motif search on the 10,000 most highly methylated CH sites. Expectedly, we recovered the TGCT motif associated with mosaic satellite repeats in both brain and liver (Ross et al., 2020), and the previously characterized TACAC-containing motif (Lister et al., 2013; de Mendoza et al., 2021), which was specific to the brain sample (Supplementary Figure 1A). Furthermore, the brain sample showed a notable enrichment in CAC trinucleotide methylation (∼1.5%) with a threefold increase compared to the unmethylated lambda genome spike-in control (∼0.5%) (Supplementary Figure 1B). This mCAC enrichment was not evident in the liver sample, thus eliminating the possibility of sequence-specific biases or artifacts pertaining to bisulfite conversion (Olova et al., 2018). We next investigated the genomic distribution of mCAC in zebrafish brains to assess if the depletion in regulatory elements and enrichment in gene bodies previously described in mammals (Lister et al., 2013; Guo et al., 2014a; He and Ecker, 2015) is evolutionarily conserved. To achieve this, we annotated transcription start sites (TSS), exons, introns, 5′UTRs, 3′UTRs, intergenic regions, and sites of H3K27ac enrichment, which correspond to active gene-regulatory elements (Kaaij et al., 2016). We found that intronic and intergenic regions are the only regions enriched in mCAC and mCH (Supplementary Figures 1C–E), whereas a notable depletion, similar to the one described in mammals, was observed at H3K27ac peaks (Supplementary Figure 1F). These results support the previous observations of mCA presence in gene bodies of vertebrate brains (de Mendoza et al., 2021) and demonstrate a conserved depletion of mCH in active regulatory regions.

**FIGURE 1:**
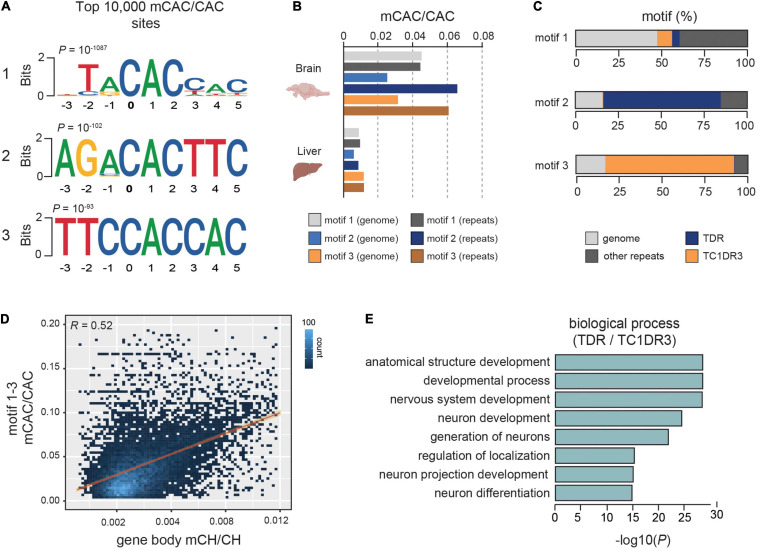
mCH is enriched at defined CAC-containing motifs in zebrafish brains. **(A)** Top three motifs called from the 10,000 most methylated CAC trinucleotides in the zebrafish brain. **(B)** Average mCAC/CAC methylation levels of the top three mCAC motifs in the bulk genome and repetitive elements of zebrafish brain and liver. **(C)** Genomic annotation of top ranked CAC motifs. **(D)** Genomic correlation between average gene body mCH/CH and mCAC/CAC at top three most methylated CAC motifs. **(E)** Gene ontology enrichment of genes containing methylated CAC motifs in TDR and TC1DR3 elements.

Further analyses of sequence motifs associated with the mCAC context unraveled two novel sequences in addition to the previously described vertebrate-conserved TACAC motif (Figure 1A). Due to previous associations of mCH with repetitive DNA in zebrafish (Ross et al., 2020), we wanted to assess if any of the most significantly methylated CAC motifs were enriched in repetitive elements. The top TACAC motif displayed comparable methylation levels in repetitive elements and in the repeat-masked genomic fraction, with an average methylation level nearly three-fold higher (∼4%) than the average global mCAC levels (Figure 1B). However, for the remaining two motifs we found a robust increase in average methylation levels at repetitive elements when compared to the repeat-masked genome (∼6.5% and 6%, Figure 1B). Further analysis revealed that the TACAC motif is broadly distributed in the genome whereas the second and third motif were mainly located in TDR and TC1DR3 repetitive elements, respectively (Figure 1C). Both TDR and TC1DR3 elements belong to the Tc1-mariner superfamily, which is found across eukaryotes. These elements are characterized by two inverted terminal repeats, and an open reading frame (ORF) (Wicker et al., 2007). The identified motifs are found on average once per element at positions 205/541 and 1005/1071 in TDR18 and TC1DR3 model sequences, respectively (Storer et al., 2021). To further validate these associations, we interrogated previously published heart and forebrain WGBS DNA methylomes (Ross et al., 2020; de Mendoza et al., 2021). These analyses again revealed brain-specific enrichment of mCH specifically at these elements (Supplementary Figure 1G), thus excluding the possibility of library preparation and sequencing artifacts causing the observed enrichment.

We also found that average methylation of the top three methylated CAC motifs correlated strongly with overall mCH in gene bodies (*R* = 0.52) (Figure 1D), suggestive of a significant contribution of CAC methylation to gene body mCH. This correlation was stronger than the one observed between average gene mCH and gene length (*R* = 0.36), which was previously described in mammals (Gabel et al., 2015; Boxer et al., 2020; Supplementary Figure 1H). Additionally, when Gene ontology (GO) analysis (Raudvere et al., 2019) of genes containing methylated TDR and TC1DR3 CAC motifs was performed, we found overrepresentation in developmental and neural development terms (Figure 1E). Due to its widespread genomic abundance the TACAC motif was omitted from the GO analysis. Overall, zebrafish brain is enriched in mCH, particularly in the CAC trinucleotide context, predominantly in introns and intergenic regions, as well as in members of the Tc1-mariner transposon family.

### mCH Is Targeted to Genes Downregulated in the Nervous System

**FIGURE 2:**
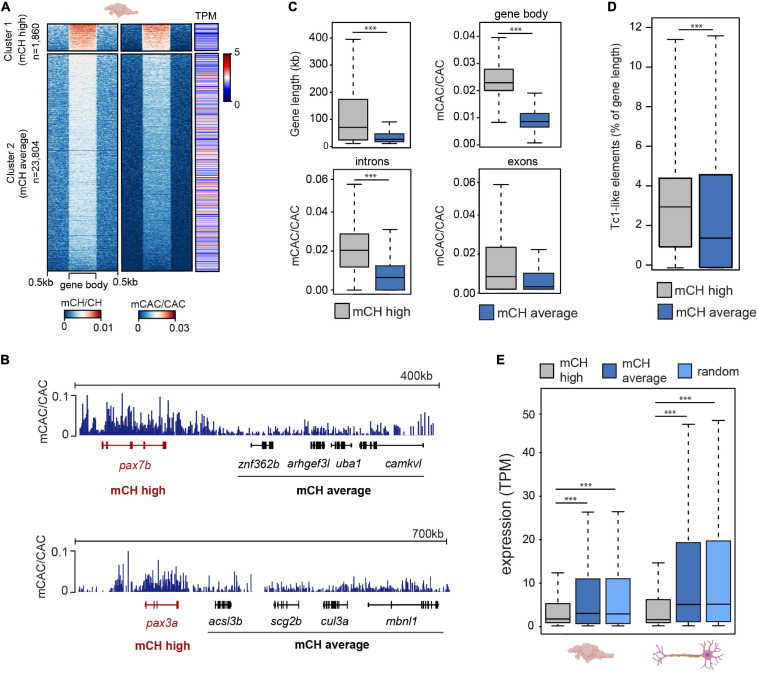
mCH is present at long genes with low expression levels. **(A)** mCH/CH levels, mCAC/CAC levels, and gene expression represented as transcripts per million (TPM) in adult zebrafish brains. **(B)** Genome browser snapshot of mCAC/CAC levels in cluster 1 (red) and cluster 2 (black) genes in adult brains. **(C)** Comparisons of gene length (top left), average gene mCAC/CAC (top right), average exon mCAC/CAC (bottom left) and average intron mCAC/CAC (bottom right) in genes with high (cluster 1) and moderate/low levels of mCAC/CAC (cluster 2) (Wilcoxon test, ∗∗∗*P* < 0.001). **(D)** Tc1-like element percentage of total gene length in genes with high (cluster 1) and moderate/low levels of mCAC/CAC (cluster 2) (Wilcoxon test, ∗∗∗*P* < 0.001). **(E)** Average expression levels (TPM) in 6-month old brains (*n* = 5) and neurons (*n* = 30) at genes with high (cluster 1), or moderate/low (cluster 2) mCH, and a random subset of genes (*n* = 1,860) sampled from cluster 2 (Wilcoxon test, ∗∗∗*P* < 0.001).

To explore the relationship between mCH and gene expression in the zebrafish brain, we plotted average gene mCH and mCAC values against gene expression (transcripts per million-TPM) levels from available datasets (Bogdanovic et al., 2016; Aramillo Irizar et al., 2018; Figure 2A). We revealed a gene cluster (*n* = 1,860) with higher-than-average mCH levels, which displayed lower expression than the bulk transcriptome (Figures 2A,B). To provide more genomic context to these findings, we first interrogated whether this elevated mCH was driven by a higher proportion of intron sequence in these genes. To that end, we plotted gene length, gene body mCAC, intron mCAC and exon mCAC for mCH-enriched genes as well as for genes that did not display any notable mCH enrichment (Figure 2C). While the mCH-enriched genes were significantly longer (Wilcoxon test, ^∗∗∗^ *P* < 0.001), in line with observations in mammals (Gabel et al., 2015; Boxer et al., 2020), the elevation in mCH was not driven exclusively by intron contribution as both introns and exons located within these genes had significantly higher levels of mCAC (Wilcoxon test, ^∗∗∗^*P* < 0.001) (Figure 2C). Interestingly, genes with higher levels of mCAC also contained a higher percentage of Tc1-like elements in relation to total gene length (Figure 2D). To confirm the observation of poorly expressed genes being marked by mCH, we plotted average TPM levels from total RNA-seq data from adult brains (Aramillo Irizar et al., 2018) and combined single cell data from neurons (Lange et al., 2020; Figure 2E). Genes with higher levels of mCH (cluster 1) had significantly lower average TPM when compared to genes with moderate/low levels of mCH (cluster 2) (Wilcoxon test, ^∗∗∗^*P* < 0.001), or to a randomly selected subset of genes (*n* = 1,860, Figure 2E) (Wilcoxon test, ^∗∗∗^*P* < 0.001). This difference in expression levels between the two clusters was even more pronounced in neurons where mCH is expected to be the highest based on mammalian data (Lister et al., 2013; Figure 2E). GO analysis of these highly mCH-methylated genes again revealed enrichment for terms associated with embryonic and neural development (Supplementary Figure 2A). This enrichment of developmental genes for mCH is also in line with observations in other vertebrate brains (de Mendoza et al., 2021).

As mCH-enriched genes are on average poorly expressed, longer, and associated with neuronal and development terms, it is yet unclear which features are most important or predictive for mCH enrichment. This is further complicated by neuronal terms and gene lengths being tightly associated in zebrafish, as discussed in our previous study (Ross et al., 2020). To further explore and rank features that may be associated with genomic mCH, we analyzed histone modification ChIP-seq data from the zebrafish brain (Yang et al., 2020) and assessed the correlations between these diverse histone marks and mCH levels in gene bodies (Supplementary Figure 2B). We also performed correlation analyses for mCH and various other genomic features (Supplementary Figure 2B). These analyses revealed that gene length has the strongest positive correlation with gene body mCH levels and that H3K4me3 at transcription start sites has the strongest negative correlation with gene body mCH. Overall, these results further demonstrate that mCH-enriched genes are longer and associated with a repressive chromatin environment. Therefore, like in mammals, mCH in the zebrafish adult brain is associated with transcriptional repression which is particularly evident in long genes.

### mCH Accumulates During Zebrafish Brain Development

**FIGURE 3:**
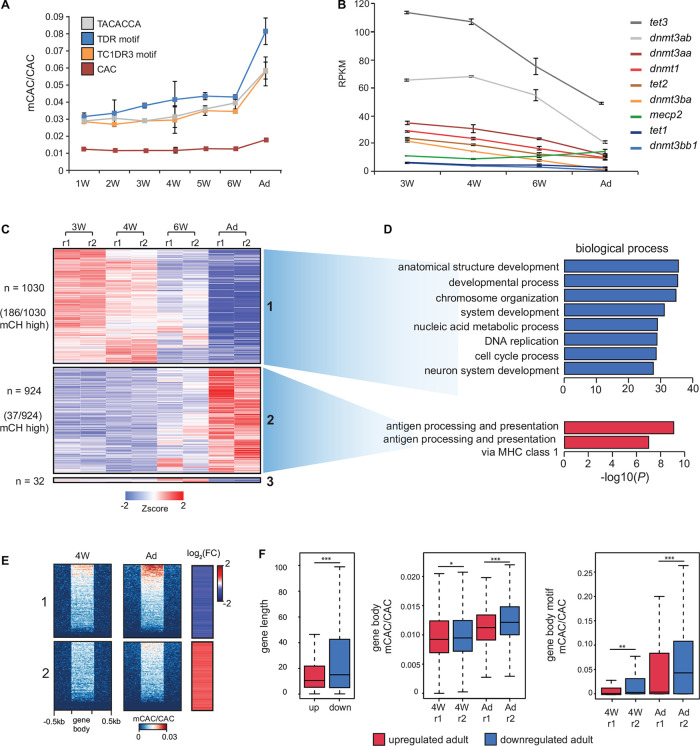
mCH accumulates in the developing nervous system. **(A)** mCAC/CAC levels at all CAC trinucleotides and top methylated CAC motifs in larval (W = weeks old) and adult (Ad) brains, as determined by RRBS. Data is represented as the average of two biological replicates with error bars (standard deviation). **(B)** RPKM (reads per kilobase per million) values of *dnmt*, *tet*, and *mecp2* transcripts in larval and adult brains determined by RNA-seq. Data is represented as the average of two biological replicates with error bars (standard deviation). **(C)** Transcription intensities of a merged collection of differentially expressed genes called between all pairwise comparisons of larval and adult stages (r1—replicate 1, *r2* = replicate 2). **(D)** Gene ontology enrichment of differentially expressed genes in larval and adult brains. **(E)** mCAC levels and relative RNA expression levels (log2 fold change 4W/Ad) at differentially expressed genes. **(F)** Comparisons of gene length (left), gene body mCAC/CAC (middle), and gene body mCAC/CAC at top methylated motifs (right) in genes that are either upregulated or downregulated in the adult brain (Wilcoxon test, ^∗^*P* < 0.05, ^∗∗^*P* < 0.01, ^∗∗∗^*P* < 0.001).

As mCH has previously been shown to accumulate during mammalian brain development (Lister et al., 2013), we next investigated whether comparable mCH dynamics could be observed in zebrafish. We generated RRBS libraries (Meissner et al., 2005) using a combination of enzymes, which were selected based on virtual digestion, to enrich for regions containing highly methylated CAC motifs (Figure 1A) identified in adult brains. We assayed zebrafish brains starting from 1 week (1W), where brain structures such as the cerebrum are not identifiable, up until 6 weeks (6W) and adulthood, where all structures are discernable (Maeyama and Nakayasu, 2000). This analysis revealed a gradual increase in mCAC in the brains of 1-week to 6-week-old zebrafish followed by a more notable increase in adult brains (Figure 3A). RNA-seq analysis across this period also revealed a gradual decrease in the expression of components of DNA methylation machinery as cells presumably become more differentiated (Figure 3B). Moreover, *mecp2* expression increased during brain development coinciding with increase in mCH. This observation supports a conserved role for MeCP2 in the regulation of genes marked by mCH in vertebrates (Guo et al., 2014a; Chen et al., 2015; Gabel et al., 2015; de Mendoza et al., 2021). Differential expression analysis of all genes across brain development revealed two major gene clusters which were either consistently upregulated (cluster 1) or downregulated (cluster 2) during brain development (Figure 3C). Interestingly, 18% of the downregulated genes belonged to the mCH-enriched gene cluster, compared to only 4% of the upregulated genes (χ^2^-test ^∗∗∗^*P* < 0.001) (Figure 3C). Downregulated genes were also associated with terms related to cell division and development while genes that were upregulated were enriched in terms associated with adaptive immunity (Figure 3D). This are consistent with ongoing developmental processes in the larval brain and with the notion that the adaptive immune system of zebrafish does not develop until 4 to 6 weeks post fertilization (Lam et al., 2004).

Since we determined that mCH dynamics in the developing zebrafish brain are comparable to developmental mCH dynamics in the postnatal mouse frontal cortex (Lister et al., 2013), we next wanted to explore if mCG dynamics similar to the one observed in mice could also be detected in zebrafish. Pairwise analyses of differentially methylated regions (DMRs) from RRBS data identified 1723 DMRs. The strongest difference in mCG was observed between 1-week-old brain and adult brain (Supplementary Figure 3A). This analysis also revealed that the 1-week-old zebrafish brain is most similar to the fetal mouse sample, as both the 1-week-old zebrafish brain and fetal mouse brain displayed most obvious differences when compared to other time points (Lister et al., 2013). The number of DMRs identified in zebrafish (*n* = 1,723) significantly differs from the number of DMRs identified in developing mouse brains (*n* > 142,835). This discrepancy is likely caused by different 5mC detection approaches (RRBS instead of WGBS), however other contributing factors could include: the use of whole brains instead of sorted neuron and glial populations, developmental stages not being directly comparable between zebrafish and mouse, adult zebrafish brains retaining more “juvenile features” such as radial glial cells and neurogenic capabilities (Schmidt et al., 2013), and different glia/neuron percentages of the samples, as mCG levels in glia have been reported to be more stable during development (Lister et al., 2013).

To understand better how methylation and gene expression dynamics track over developmental time, we generated WGBS datasets for 4-week-old brain tissue and compared these data against adult brain WGBS and RNA-seq data. Analysis of mCAC levels of differentially expressed genes revealed that developmentally downregulated genes accumulate more mCH when compared to upregulated ones (Figure 3E). This trend was also observed when visualizing mCAC levels and gene expression levels across all genes (Supplementary Figures 3B,C). Furthermore, quantification of mCAC levels at all mCAC trinucleotides and at the highly methylated motifs (all three combined), confirmed a significant increase in the methylation of developmentally downregulated genes (Wilcoxon test, ^∗∗∗^*P* < 0.001) (Figure 3F). This increase in mCH in adult brains is uncoupled from global mCG changes, as DMR analysis of these WGBS datasets revealed 23,992 DMRs with the majority (*n* = 18,522) becoming hypomethylated during nervous system development (Supplementary Figures 3D,E). Altogether, these results demonstrate robust anticorrelation between mCH and gene expression during brain development in zebrafish, as well as developmental mCH accumulation, in line with observations in mammals.

### Dnmt3a Enzymes Are Required for Methylation of CAC Trinucleotides in the Zebrafish Brain

**FIGURE 4:**
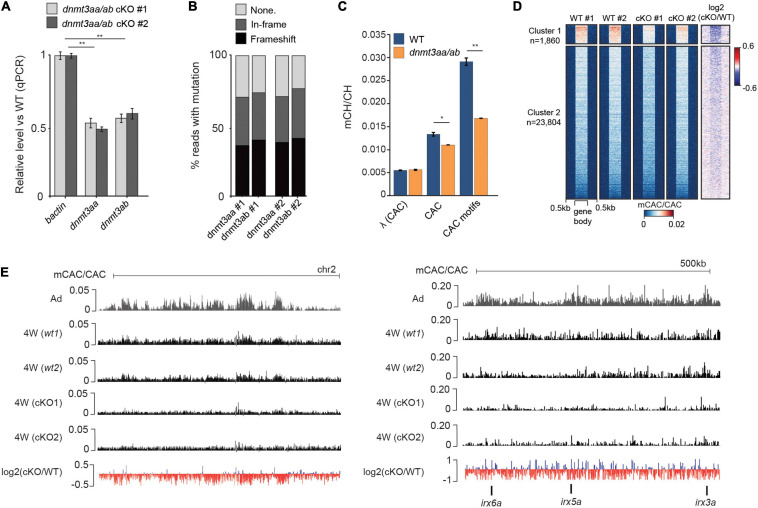
Dnmt3a enzymes are required for methylation of CAC trinucleotides in the zebrafish brain. **(A)** Transcript levels of *dnmt3aa* and *dnmt3ab* in 4-week-old *dnmt3aa/ab* CRISPR/Cas9 KO (cKO) zebrafish brains relative to wild type (WT). The data is represented as the mean of technical replicates with error bars showing the standard error (two sample *t*-test, **P < 0.01). **(B)** Percentage of reads with no mutation, in-frame mutations, or frameshift mutations, which map to *dnmt3aa* and *dnmt3ab* loci in *dnmt3aa/ab* cKOs. **(C)** Average mCAC/CAC levels at all CAC trinucleotides and the top methylated CAC motifs in 4-week-old WT and *dnmt3aa/ab* cKO brain. The data is represented as the average of two WGBS biological replicates (Wilcoxon test, *P < 0.05, **P < 0.01, λ = unmethylated lambda spike in control). **(D)** mCAC/CAC levels of all gene bodies in 4-week-old WT and *dnmt3aa/ab* cKO brains. **(E)** Genome browser snapshot of mCAC/CAC levels in adult brains, 4-week-old WT brains and 4-week-old *dnmt3aa/ab* cKO brains.

Finally, to investigate if Dnmt3a-dependent methylation of CAC trinucleotides is evolutionarily conserved in zebrafish, we generated *dnmt3aa*/*dnmt3ab* CRISPR/Cas9 double knockouts (cKO). qPCR analysis of cDNA extracted from brains of 4-week-old cKOs revealed their partial knockout status with a 50% reduction in expression levels for both *dnmt3aa* and *dnmt3ab* (Figure 4A). Sequencing of amplicons surrounding the CRISPR/Cas9 cut sites demonstrated comparable estimates of genome editing efficiency (Figure 4B). Finally, WGBS analysis of these samples demonstrated that depletion of *dnmt3aa*/*dnmt3ab* resulted in a significant (*P* < 0.05, Wilcoxon test) reduction in global mCAC levels as well as in a notable reduction (43%) of mCH at top mCAC motifs (*P* < 0.01, Wilcoxon test) (Figure 4C). This significant reduction in mCH observed already in these partial cKOs, which could have undergone possible selection for more wild type cells, suggests that Dnmt3aa and Dnmt3ab are the major enzymes responsible for neural mCAC deposition. The reduction in mCAC levels in these cKOs can be observed across the majority of gene bodies (Figure 4D) and on a genome-wide and locus-specific scale (Figure 4E). Notably, these perturbations in mCH did not result in any obvious morphological defects in the cKO fish (data not shown). Finer analysis of brain morphology, behavior, and potential inflammatory processes in these animals will be a focus of future studies. Additionally, no changes in global CpG methylation levels or significant DMRs could be detected (Supplementary Figure 3F) between the cKO and WT brain, suggestive of mCH deposition being a major function of Dnmt3a enzymes in zebrafish. This is contrary to what has been observed in mouse neurons, where Dnmt3a KO resulted in ∼10% decrease in global mCG/CG levels (Lavery et al., 2020). However, given the incomplete KO, the possible redundancies with other zebrafish DNMTs (Goll and Halpern, 2011), the propagation by Dnmt1 once established, and the use of bulk cell DNA methylome data, we cannot completely rule out a role for these enzymes in neuronal CpG methylation. Nevertheless, the clear role in mCH deposition, and the fact that Dnmt3a enzymes can be traced back to the root of vertebrates (de Mendoza et al., 2021), suggests conserved functional importance of mCH in the vertebrate nervous system.

## Discussion

mCH has been established as an important base modification with likely biological functions during mammalian brain development (Lister et al., 2013), and links to Rett syndrome pathogenesis (Chen et al., 2015; Gabel et al., 2015; Boxer et al., 2020; Lavery et al., 2020), there are still many unknowns related to its regulation and function. Furthermore, the evolutionary conservation of mCH, the mCH “writer”—DNM3TA, and the mCH “reader”—MeCP2 in vertebrates suggests that these regulatory pathways could have an ancestral role in vertebrate neurobiology (de Mendoza et al., 2021). In the current manuscript, we describe the evolutionary conservation of developmental mCH dynamics in the zebrafish nervous system. In zebrafish, like in mammals, mCH is enriched at CAC trinucleotides in gene bodies where it accumulates during brain development. Also, in line with observations in mammals, mCH depletion is evident at H3K4me3- and H3K27ac-marked regulatory regions. Similarly to our recent work on TGCT methylation of mosaic satellite repeats in zebrafish (Ross et al., 2020), and previous reports of mCH enrichment at repetitive elements in mammals (Ziller et al., 2011; Arand et al., 2012; Guo et al., 2014b), we find high levels of mCH at defined motifs associated with Tc1-like transposons (∼6%). This recurring observation of mCH enrichment at repeats in multiple species supports a possible role for mCH, or DNMT3A recruitment, in the regulation of repetitive elements. In mammalian brains, active transposition of repeat elements has been shown to drive mosaicism in neuronal genomes (Muotri et al., 2005; Macia et al., 2017; Bodea et al., 2018), while MeCP2 was described as a repressor of LINE-1 elements in mouse neurons (Yu et al., 2001; Muotri et al., 2010). These data thus suggest that mCH could play an important role in regulating repetitive elements in the vertebrate brain, particularly at CG-sparse regions or active repeats such as Tc1-like transposons. These observations are also reminiscent of mCH targeting by DNMT3-related plant enzymes (Law and Jacobsen, 2010) and the findings that mCH plays vital roles in transposon silencing in plants (Domb et al., 2020).

Finally, in the current study, we generate transient CRISRPR/Cas9 KOs for *dnmt3aa*/*dnmt3ab* and demonstrate a conserved role for these enzymes in the deposition of mCH, and mCAC in particular, in the zebrafish brain. These partial KOs only have an obvious effect on mCH but not mCG levels, suggestive of a direct conservation for mCH in the vertebrate nervous system. Overall, this work provides novel insight into the evolutionary conservation of vertebrate mCH patterning and highlights the utility of the zebrafish model system, which is amenable to CRISPR/Cas9 screens, drug screens and developmental imaging, for the studies of mCH and brain development *in vivo*.

## Data Availability Statement

Data generated for this submission have been uploaded to ArrayExpress https://www.ebi.ac.uk/arrayexpress/ under the accession number E-MTAB-9924.

## Ethics Statement

The animal study was reviewed and approved by the Garvan Institute of Medical Research Animal Ethics Committee (AEC approval 20/09).

## Author Contributions

OB conceived the study. SR performed bioinformatic analyses and CRISPR/Cas9 experiments. DH extracted brain samples. SR and OB wrote the manuscript. All authors contributed to read, and approved the final manuscript.

## Conflict of Interest

The authors declare that the research was conducted in the absence of any commercial or financial relationships that could be construed as a potential conflict of interest.

## References

[B1] Aramillo Irizar P. Schäuble S. Esser D. Groth M. Frahm C. Priebe S. (2018). Transcriptomic alterations during ageing reflect the shift from cancer to degenerative diseases in the elderly. *Nat. Commun.* 9:327. 10.1038/s41467-017-02395-2 29382830 PMC5790807

[B2] Arand J. Spieler D. Karius T. Branco M. R. Meilinger D. Meissner A. (2012). In vivo control of CpG and non-CpG DNA methylation by DNA methyltransferases. *PLoS Genet.* 8:e1002750. 10.1371/journal.pgen.1002750 22761581 PMC3386304

[B3] Bird A. (2002). DNA methylation patterns and epigenetic memory. *Genes Dev.* 16 6–21. 10.1101/gad.947102 11782440

[B4] Bodea G. O. McKelvey E. G. Z. Faulkner G. J. (2018). Retrotransposon-induced mosaicism in the neural genome. *Open Biol.* 8:180074. 10.1098/rsob.180074 30021882 PMC6070720

[B5] Bogdanovic O. Smits A. H. de la Calle Mustienes E. Tena J. J. Ford E. Williams R. (2016). Active DNA demethylation at enhancers during the vertebrate phylotypic period. *Nat. Genet.* 48 417–426. 10.1038/ng.3522 26928226 PMC5912259

[B6] Bolger A. M. Lohse M. Usadel B. (2014). Trimmomatic: a flexible trimmer for Illumina sequence data. *Bioinformatics* 30 2114–2120. 10.1093/bioinformatics/btu170 24695404 PMC4103590

[B7] Boxer L. D. Renthal W. Greben A. W. Whitwam T. Silberfeld A. Stroud H. (2020). MeCP2 Represses the Rate of Transcriptional Initiation of Highly Methylated Long Genes. *Mol. Cell* 77 294.e–309.e. 10.1016/j.molcel.2019.10.032 31784358 PMC6982532

[B8] Chen H. Smith A. D. Chen T. (2016). WALT: fast and accurate read mapping for bisulfite sequencing. *Bioinformatics* 32 3507–3509. 10.1093/bioinformatics/btw490 27466624 PMC5181568

[B9] Chen L. Chen K. Lavery L. A. Baker S. A. Shaw C. A. Li W. (2015). MeCP2 binds to non-CG methylated DNA as neurons mature, influencing transcription and the timing of onset for Rett syndrome. *Proc. Natl. Acad. Sci. U. S. A.* 112 5509–5514. 10.1073/pnas.1505909112 25870282 PMC4418849

[B10] Clemens A. W. Wu D. Y. Moore J. R. Christian D. L. Zhao G. Gabel H. W. (2020). MeCP2 Represses Enhancers through Chromosome Topology-Associated DNA Methylation. *Mol. Cell* 77 279.e–293.e. 10.1016/j.molcel.2019.10.033 31784360 PMC6980697

[B11] de Mendoza A. Lister R. Bogdanovic O. (2019). Evolution of DNA Methylome Diversity in Eukaryotes. *J. Mol. Biol.* 2019:003. 10.1016/j.jmb.2019.11.003 31726061

[B12] de Mendoza A. Poppe D. Buckberry S. Pflueger J. Albertin C. Daish T. (2021). The emergence of neural non-CpG methylation system in vertebrates. *Nat. Ecol. Evol.* 2021 1371–1372. 10.1038/s41559-020-01371-2 33462491 PMC7116863

[B13] Dobin A. Davis C. A. Schlesinger F. Drenkow J. Zaleski C. Jha S. (2013). STAR: ultrafast universal RNA-seq aligner. *Bioinformatics* 29 15–21. 10.1093/bioinformatics/bts635 23104886 PMC3530905

[B14] Domb K. Katz A. Harris K. Yaari R. Kaisler E. Nguyen V. (2020). DNA methylation mutants in *Physcomitrella patens* elucidate individual roles of CG and non-CG methylation in genome regulation. *PNAS*. 117 33700–33710. 10.1073/pnas.2011361117 33376225 PMC7777129

[B15] Feng H. Conneely K. Wu H. (2014). A bayesian hierarchical model to detect differentially methylated loci from single nucleotide resolution sequencing data. *Nucleic Acids Res.* 2014:154. 10.1093/nar/gku154 24561809 PMC4005660

[B16] Gabel H. W. Kinde B. Stroud H. Gilbert C. S. Harmin D. A. Kastan N. R. (2015). Disruption of DNA-methylation-dependent long gene repression in Rett syndrome. *Nature* 522 89–93. 10.1038/nature14319 25762136 PMC4480648

[B17] Gao H. Bu Y. Wu Q. Wang X. Chang N. Lei L. (2015). Mecp2 regulates neural cell differentiation by suppressing the Id1 to Her2 axis in zebrafish. *J. Cell Sci.* 128 2340–2350. 10.1242/jcs.167874 25948585

[B18] Goll M. G. Halpern M. E. (2011). DNA methylation in zebrafish. *Prog. Mol. Biol. Transl. Sci.* 101 193–218. 10.1016/B978-0-12-387685-0.00005-6 21507352 PMC5455991

[B19] Guo J. U. Su Y. Shin J. H. Shin J. Li H. Xie B. (2014a). Distribution, recognition and regulation of non-CpG methylation in the adult mammalian brain. *Nat. Neurosci.* 17 215–222. 10.1038/nn.3607 24362762 PMC3970219

[B20] Guo W. Chung W.-Y. Qian M. Pellegrini M. Zhang M. Q. (2014b). Characterizing the strand-specific distribution of non-CpG methylation in human pluripotent cells. *Nucleic Acids Res.* 42 3009–3016. 10.1093/nar/gkt1306 24343027 PMC3950701

[B21] Guo W. Zhu P. Pellegrini M. Zhang M. Q. Wang X. Ni Z. (2018). CGmapTools improves the precision of heterozygous SNV calls and supports allele-specific methylation detection and visualization in bisulfite-sequencing data. *Bioinformatics* 34 381–387. 10.1093/bioinformatics/btx595 28968643 PMC6454434

[B22] He Y. Ecker J. R. (2015). Non-CG Methylation in the Human Genome. *Annu. Rev. Genomics Hum. Genet.* 16 55–77. 10.1146/annurev-genom-090413-025437 26077819 PMC4729449

[B23] Heinz S. Benner C. Spann N. Bertolino E. Lin Y. C. Laslo P. (2010). Simple Combinations of Lineage-Determining Transcription Factors Prime cis-Regulatory Elements Required for Macrophage and B Cell Identities. *Mole. Cell* 38 576–589. 10.1016/j.molcel.2010.05.004 20513432 PMC2898526

[B24] Jones P. A. (2012). Functions of DNA methylation: islands, start sites, gene bodies and beyond. *Nat. Rev. Genet.* 13 484–492. 10.1038/nrg3230 22641018

[B25] Kaaij L. J. T. Mokry M. Zhou M. Musheev M. Geeven G. Melquiond A. S. J. (2016). Enhancers reside in a unique epigenetic environment during early zebrafish development. *Genome Biol.* 17:146. 10.1186/s13059-016-1013-1 27381023 PMC4934011

[B26] Kent W. J. Sugnet C. W. Furey T. S. Roskin K. M. Pringle T. H. Zahler A. M. (2002). The human genome browser at UCSC. *Genome Res.* 12 996–1006. 10.1101/gr.229102 12045153 PMC186604

[B27] Lam S. H. Chua H. L. Gong Z. Lam T. J. Sin Y. M. (2004). Development and maturation of the immune system in zebrafish, Danio rerio: a gene expression profiling, in situ hybridization and immunological study. *Dev. Comp. Immunol.* 28 9–28. 10.1016/s0145-305x(03)00103-4 12962979

[B28] Lange C. Rost F. Machate A. Reinhardt S. Lesche M. Weber A. (2020). Single cell sequencing of radial glia progeny reveals the diversity of newborn neurons in the adult zebrafish brain. *Development* 147:185595. 10.1242/dev.185595 31908317 PMC6983714

[B29] Langmead B. Salzberg S. L. (2012). Fast gapped-read alignment with Bowtie 2. *Nat. Methods* 9 357–359. 10.1038/nmeth.1923 22388286 PMC3322381

[B30] Lavery L. A. Ure K. Wan Y.-W. Luo C. Trostle A. J. Wang W. (2020). Losing Dnmt3a dependent methylation in inhibitory neurons impairs neural function by a mechanism impacting Rett syndrome. *Elife* 9:52981. 10.7554/eLife.52981 32159514 PMC7065908

[B31] Law J. A. Jacobsen S. E. (2010). Establishing, maintaining and modifying DNA methylation patterns in plants and animals. *Nat. Rev. Genet.* 11 204–220. 10.1038/nrg2719 20142834 PMC3034103

[B32] Lister R. Mukamel E. A. Nery J. R. Urich M. Puddifoot C. A. Johnson N. D. (2013). Global epigenomic reconfiguration during mammalian brain development. *Science* 341:1237905. 10.1126/science.1237905 23828890 PMC3785061

[B33] Lister R. Pelizzola M. Dowen R. H. Hawkins R. D. Hon G. Tonti-Filippini J. (2009). Human DNA methylomes at base resolution show widespread epigenomic differences. *Nature* 462 315–322. 10.1038/nature08514 19829295 PMC2857523

[B34] Macia A. Widmann T. J. Heras S. R. Ayllon V. Sanchez L. Benkaddour-Boumzaouad M. (2017). Engineered LINE-1 retrotransposition in nondividing human neurons. *Genome Res.* 27 335–348. 27965292 10.1101/gr.206805.116 PMC5340962

[B35] Maeyama K. Nakayasu H. (2000). Postembryonic Neurogenesis in Zebrafish (Danio rerio) Brain: Presence of Two Different Systems. J*zoo* 17 959–966. 10.2108/zsj.17.959

[B36] Meissner A. Gnirke A. Bell G. W. Ramsahoye B. Lander E. S. Jaenisch R. (2005). Reduced representation bisulfite sequencing for comparative high-resolution DNA methylation analysis. *Nucleic Acids Res.* 33 5868–5877. 10.1093/nar/gki901 16224102 PMC1258174

[B37] Moreno-Mateos M. A. Vejnar C. E. Beaudoin J.-D. Fernandez J. P. Mis E. K. Khokha M. K. (2015). CRISPRscan: designing highly efficient sgRNAs for CRISPR-Cas9 targeting in vivo. *Nat. Methods* 12 982–988. 26322839 10.1038/nmeth.3543 PMC4589495

[B38] Muotri A. R. Chu V. T. Marchetto M. C. N. Deng W. Moran J. V. Gage F. H. (2005). Somatic mosaicism in neuronal precursor cells mediated by L1 retrotransposition. *Nature* 435 903–910. 10.1038/nature03663 15959507

[B39] Muotri A. R. Marchetto M. C. N. Coufal N. G. Oefner R. Yeo G. Nakashima K. (2010). L1 retrotransposition in neurons is modulated by MeCP2. *Nature* 468 443–446. 10.1038/nature09544 21085180 PMC3059197

[B40] Nozawa K. Lin Y. Kubodera R. Shimizu Y. Tanaka H. Ohshima T. (2017). Zebrafish Mecp2 is required for proper axonal elongation of motor neurons and synapse formation. *Dev. Neurobiol.* 77 1101–1113. 10.1002/dneu.22498 28371371

[B41] Olova N. Krueger F. Andrews S. Oxley D. Berrens R. V. Branco M. R. (2018). Comparison of whole-genome bisulfite sequencing library preparation strategies identifies sources of biases affecting DNA methylation data. *Genome Biol.* 19:33. 10.1186/s13059-018-1408-2 29544553 PMC5856372

[B42] Pietri T. Roman A.-C. Guyon N. Romano S. A. Washbourne P. Moens C. B. (2013). The first mecp2-null zebrafish model shows altered motor behaviors. *Front. Neural Circuits* 7:118. 10.3389/fncir.2013.00118 23874272 PMC3712905

[B43] Pinello L. Canver M. C. Hoban M. D. Orkin S. H. Kohn D. B. Bauer D. E. (2016). Analyzing CRISPR genome-editing experiments with CRISPResso. *Nat. Biotechnol.* 34 695–697. 10.1038/nbt.3583 27404874 PMC5242601

[B44] Quinlan A. R. Hall I. M. (2010). BEDTools: a flexible suite of utilities for comparing genomic features. *Bioinformatics* 26 841–842. 10.1093/bioinformatics/btq033 20110278 PMC2832824

[B45] Raman A. T. Pohodich A. E. Wan Y.-W. Yalamanchili H. K. Lowry W. E. Zoghbi H. Y. (2018). Apparent bias toward long gene misregulation in MeCP2 syndromes disappears after controlling for baseline variations. *Nat. Commun.* 9:3225. 10.1038/s41467-018-05627-1 30104565 PMC6089998

[B46] Ramirez F. Dundar F. Diehl S. Gruning B. A. Manke T. (2014). deepTools: a flexible platform for exploring deep-sequencing data. *Nucleic Acids Res.* 42 W187–W191. 10.1093/nar/gku365 24799436 PMC4086134

[B47] Raudvere U. Kolberg L. Kuzmin I. Arak T. Adler P. Peterson H. (2019). g:Profiler: a web server for functional enrichment analysis and conversions of gene lists (2019 update). *Nucleic Acids Res.* 47 W191–W198. 10.1093/nar/gkz369 31066453 PMC6602461

[B48] Robinson J. T. Thorvaldsdóttir H. Winckler W. Guttman M. Lander E. S. Getz G. (2011). Integrative genomics viewer. *Nat. Biotechnol.* 29 24–26. 10.1038/nbt.1754 21221095 PMC3346182

[B49] Robinson M. D. McCarthy D. J. Smyth G. K. (2010). edgeR: a Bioconductor package for differential expression analysis of digital gene expression data. *Bioinformatics* 26 139–140. 10.1093/bioinformatics/btp616 19910308 PMC2796818

[B50] Ross S. E. Angeloni A. Geng F.-S. de Mendoza A. Bogdanovic O. (2020). Developmental remodelling of non-CG methylation at satellite DNA repeats. *Nucleic Acids Res.* 2020 1135. 10.1093/nar/gkaa1135 33271598 PMC7736785

[B51] Schmidt R. Strähle U. Scholpp S. (2013). Neurogenesis in zebrafish – from embryo to adult. *Neural. Dev.*. 2013:3. 10.1186/1749-8104-8-3 23433260 PMC3598338

[B52] Schübeler D. (2015). Function and information content of DNA methylation. *Nature* 517 321–326. 10.1038/nature14192 25592537

[B53] Schultz M. D. He Y. Whitaker J. W. Hariharan M. Mukamel E. A. Leung D. (2015). Human body epigenome maps reveal noncanonical DNA methylation variation. *Nature* 523 212–216. 10.1038/nature14465 26030523 PMC4499021

[B54] Skvortsova K. Tarbashevich K. Stehling M. Lister R. Irimia M. Raz E. (2019). Retention of paternal DNA methylome in the developing zebrafish germline. *Nat. Commun.* 10 10895–10896. 10.1038/s41467-019-10895-6 31296860 PMC6624265

[B55] Storer J. Hubley R. Rosen J. (2021). The Dfam community resource of transposable element families, sequence models, and genome annotations. *Mobile DNA* 2021:00230. 10.1186/s13100-020-00230-y 33436076 PMC7805219

[B56] Tarasov A. Vilella A. J. Cuppen E. Nijman I. J. Prins P. (2015). Sambamba: fast processing of NGS alignment formats. *Bioinformatics* 31 2032–2034. 10.1093/bioinformatics/btv098 25697820 PMC4765878

[B57] van der Vaart M. Svoboda O. Weijts B. G. Espín-Palazón R. Sapp V. Pietri T. (2017). Mecp2 regulates tnfa during zebrafish embryonic development and acute inflammation. *Dis. Model. Mech.* 10 1439–1451. 10.1242/dmm.026922 28993314 PMC5769600

[B58] Wagih O. (2017). ggseqlogo: a versatile R package for drawing sequence logos. *Bioinformatics* 33 3645–3647. 10.1093/bioinformatics/btx469 29036507

[B59] Wicker T. Sabot F. Hua-Van A. Bennetzen J. L. Capy P. Chalhoub B. (2007). A unified classification system for eukaryotic transposable elements. *Nat. Rev. Genet.* 8 973–982. 10.1038/nrg2165 17984973

[B60] Yang H. Luan Y. Liu T. Lee H. Fang L. Wang Y. (2020). A map of *cis*-regulatory elements and 3D genome structures in zebrafish. *Nature* 588 337–343. 10.1038/s41586-020-2962-9 33239788 PMC8183574

[B61] Yu F. Zingler N. Schumann G. Strätling W. H. (2001). Methyl-CpG-binding protein 2 represses LINE-1 expression and retrotransposition but not Alu transcription. *Nucleic Acids Res.* 29 4493–4501. 10.1093/nar/29.21.4493 11691937 PMC60185

[B62] Zhang H. Lang Z. Zhu J.-K. (2018). Dynamics and function of DNA methylation in plants. *Nat. Rev. Mol. Cell Biol.* 19 489–506. 10.1038/s41580-018-0016-z 29784956

[B63] Zhang Y. Liu T. Meyer C. A. Eeckhoute J. Johnson D. S. Bernstein B. E. (2008). Model-based analysis of ChIP-Seq (MACS). *Genome Biol.* 9:R137. 10.1186/gb-2008-9-9-r137 18798982 PMC2592715

[B64] Ziller M. J. Muller F. Liao J. Zhang Y. Gu H. Bock C. (2011). Genomic distribution and inter-sample variation of non-CpG methylation across human cell types. *PLoS Genet.* 7:e1002389. 10.1371/journal.pgen.1002389 22174693 PMC3234221

