# Supplementary material for: Developmental Accumulation of Gene Body and Transposon Non-CpG Methylation in the Zebrafish Brain
Source: Front Cell Dev Biol. 2021 Mar 4;9:643603. doi: 10.3389/fcell.2021.643603 (PMC7978034; doi:10.3389/fcell.2021.643603)
Supplement: Supplementary file 2 [file Image_1.pdf]

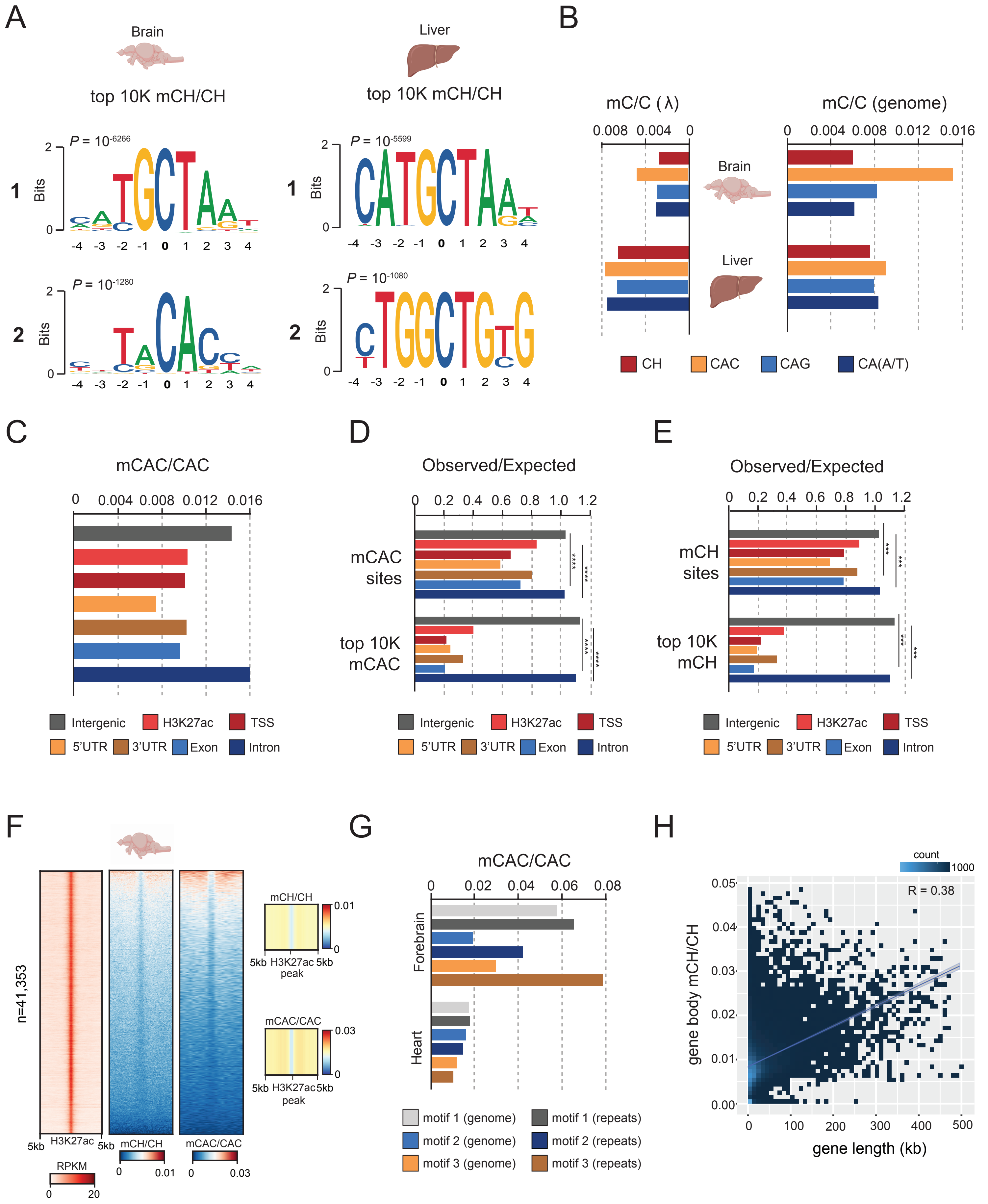

Supplementary Figure 1. mCH is enriched at defined CAC-containing motifs in zebrafish brains. A) Top two motifs called from 10,000 most highly methylated CH dinucleotides in zebrafish brain and liver. **B)** Average non-CG methylation levels (mC/C) at CH, CAC, CAG and CA(A/T) nucleotides in brain, liver and unmethylated lambda spike-in controls (λ). **C)** Average CAC methylation levels across diverse genomic features in the zebrafish brain. **D)** Observed-over-expected ratios of all methylated CAC sites and the 10,000 most highly methylated CAC sites (χ^2 test *** $P < 0.001$). **E)** Observed-over-expected ratios of all methylated CH sites and the 10,000 most highly methylated CH sites (χ^2 test *** $P < 0.001$). **F)** H3K27ac enrichment expressed as reads per kilobase per million (RPKM), mCH/CH and mCAC/CAC in H3K27ac peaks in adult zebrafish brains. **G)** Average mCAC/CAC methylation levels of the top three mCAC motifs in the bulk genome and repetitive elements of zebrafish forebrain and heart. **H)** Genome-wide correlation between average gene body mCAC/CAC and gene length.
