# Supplementary material for: Developmental Accumulation of Gene Body and Transposon Non-CpG Methylation in the Zebrafish Brain
Source: Front Cell Dev Biol. 2021 Mar 4;9:643603. doi: 10.3389/fcell.2021.643603 (PMC7978034; doi:10.3389/fcell.2021.643603)
Supplement: Supplementary file 3 [file Image_2.pdf]

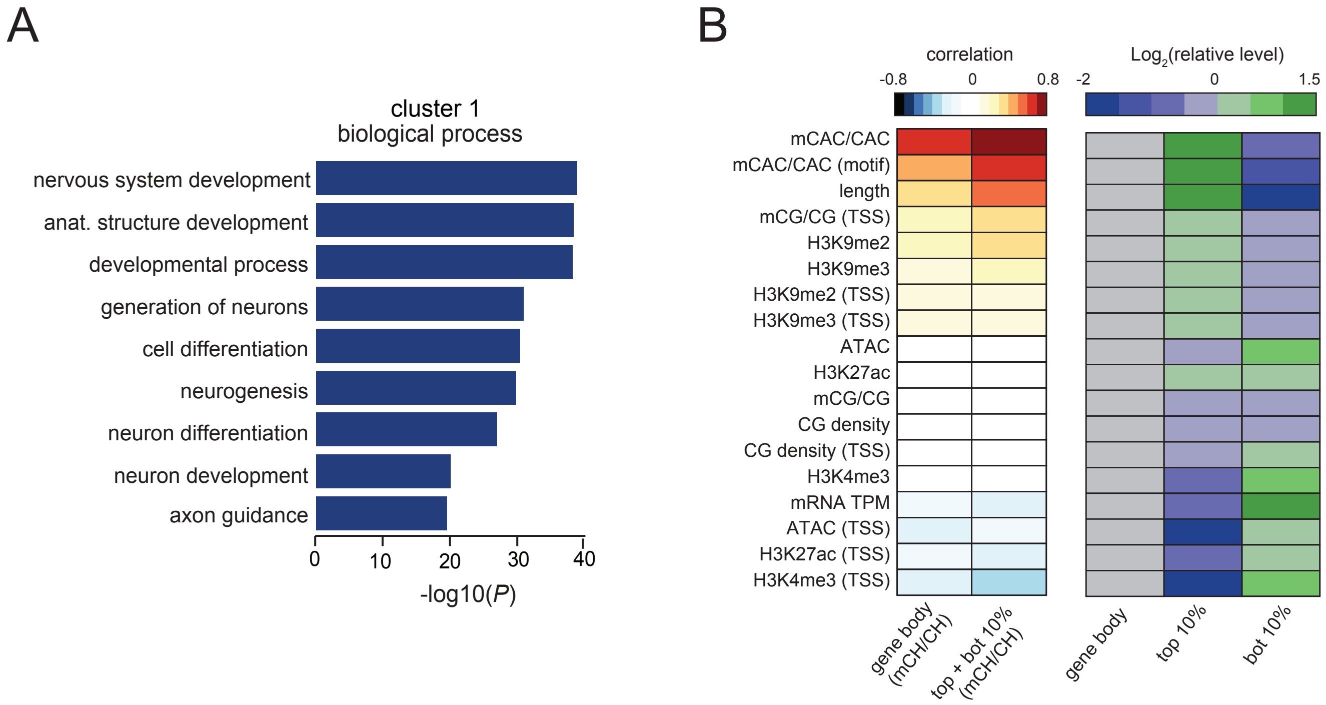

Supplementary Figure 2. mCH is enriched at long and repressed genes. A) Gene ontology enrichment of genes with high levels of mCAC/CAC. **B)** Spearman correlation coefficients between diverse genomic and epigenomic features and gene body mCH/CH levels at all genes and the top and bottom 10% of CH methylated genes (left). Log2(relative levels) of diverse genomic and epigenomic features at all genes and the top and bottom 10% of CH methylated genes (right).
