# Supplementary material for: Developmental Accumulation of Gene Body and Transposon Non-CpG Methylation in the Zebrafish Brain
Source: Front Cell Dev Biol. 2021 Mar 4;9:643603. doi: 10.3389/fcell.2021.643603 (PMC7978034; doi:10.3389/fcell.2021.643603)
Supplement: Supplementary file 4 [file Image_3.pdf]

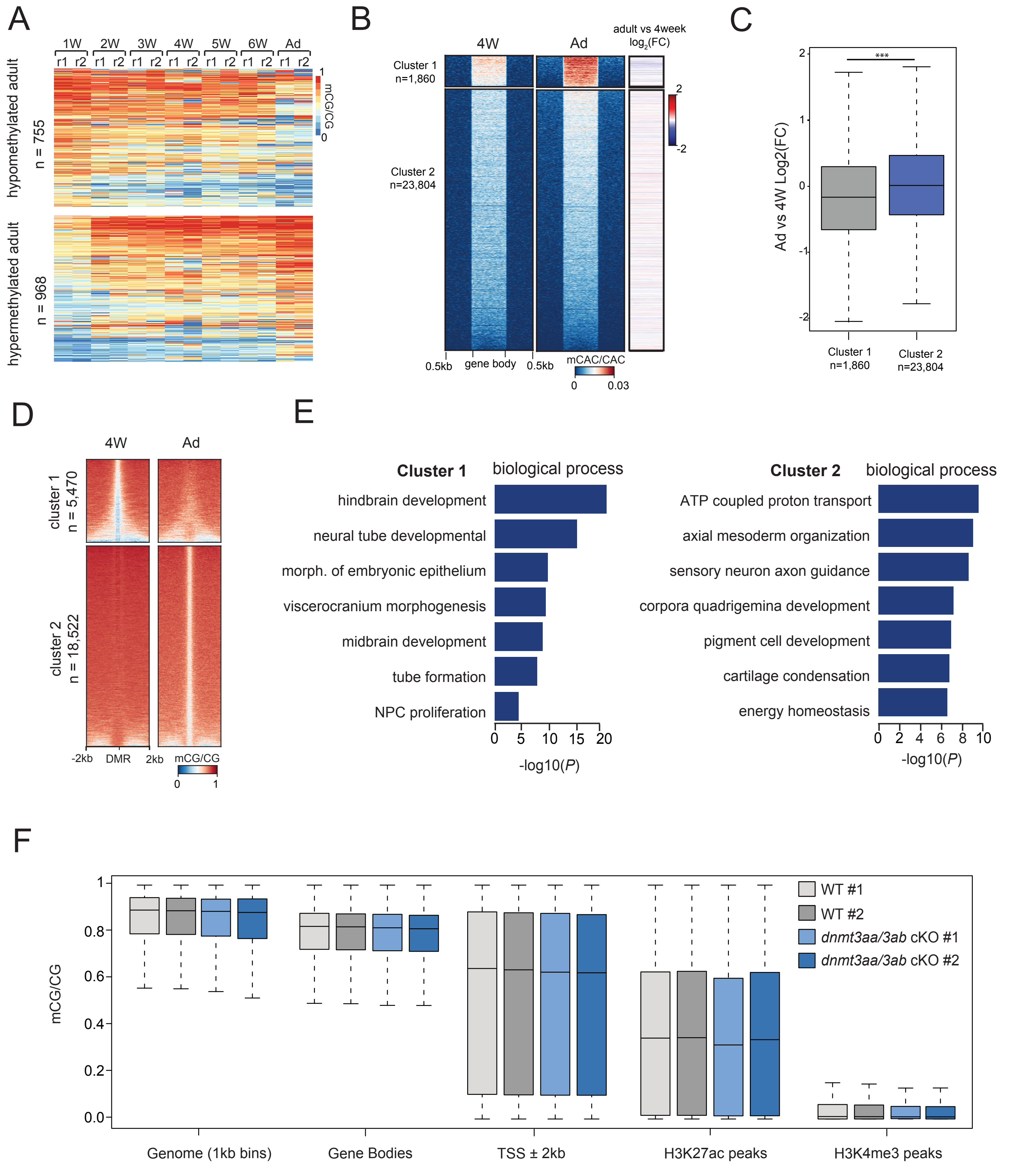

Supplementary Figure 3. DNA methylation and gene expression dynamics during zebrafish brain development. **A)** mCG/CG methylation levels at differentially methylated regions (DMRs) in larval (W = weeks old) and adult (Ad) brains, as determined by RRBS. DMRs were split into hypomethylated or hypermethylated in adult vs one-week, and hierarchically clustered. **B)** mCAC levels and relative RNA expression levels (log2 fold change Ad/4w) at all genes. Positive fold change indicates upregulation in adult. **C)** RNA expression levels in adult and four-week-old brains (log2 fold change Ad/4w) in genes marked by high levels of mCH (cluster 1) vs other genes (cluster 2) (Wilcoxon test, *** $P < 0.001$). Positive fold change indicates upregulation in adult. **D)** mCG/CG methylation levels at differentially methylated regions (DMRs) in four-week-old (4W) and adult (Ad) brain samples. **E)** GO enrichment of hypomethylated regions in four-week-old brains (cluster 1) and hypomethylated regions in adult brains (cluster 2). **F)** mCG/CG methylation levels in genomic and epigenomic features in four-week-old WT brains and four-week-old *dnmt3aa/ab* cKO brains.
